# K70Q Adds High-Level Tenofovir Resistance to “Q151M Complex” HIV Reverse Transcriptase through the Enhanced Discrimination Mechanism

**DOI:** 10.1371/journal.pone.0016242

**Published:** 2011-01-13

**Authors:** Atsuko Hachiya, Eiichi N. Kodama, Matthew M. Schuckmann, Karen A. Kirby, Eleftherios Michailidis, Yasuko Sakagami, Shinichi Oka, Kamalendra Singh, Stefan G. Sarafianos

**Affiliations:** 1 Department of Molecular Microbiology and Immunology, University of Missouri School of Medicine, Columbia, Missouri, United States of America; 2 AIDS Clinical Center, National Center for Global Health and Medicine, Tokyo, Japan; 3 Division of Emerging Infectious Diseases, Tohoku University School of Medicine, Sendai, Japan; 4 Institute for Virus Research, Kyoto University, Kyoto, Japan; University of Pittsburgh, United States of America

## Abstract

HIV-1 carrying the “Q151M complex” reverse transcriptase (RT) mutations (A62V/V75I/F77L/F116Y/Q151M, or Q151Mc) is resistant to many FDA-approved nucleoside RT inhibitors (NRTIs), but has been considered susceptible to tenofovir disoproxil fumarate (TFV-DF or TDF). We have isolated from a TFV-DF-treated HIV patient a Q151Mc-containing clinical isolate with high phenotypic resistance to TFV-DF. Analysis of the genotypic and phenotypic testing over the course of this patient's therapy lead us to hypothesize that TFV-DF resistance emerged upon appearance of the previously unreported K70Q mutation in the Q151Mc background. Virological analysis showed that HIV with only K70Q was not significantly resistant to TFV-DF. However, addition of K70Q to the Q151Mc background significantly enhanced resistance to several approved NRTIs, and also resulted in high-level (10-fold) resistance to TFV-DF. Biochemical experiments established that the increased resistance to tenofovir is not the result of enhanced excision, as K70Q/Q151Mc RT exhibited diminished, rather than enhanced ATP-based primer unblocking activity. Pre-steady state kinetic analysis of the recombinant enzymes demonstrated that addition of the K70Q mutation selectively decreases the binding of tenofovir-diphosphate (TFV-DP), resulting in reduced incorporation of TFV into the nascent DNA chain. Molecular dynamics simulations suggest that changes in the hydrogen bonding pattern in the polymerase active site of K70Q/Q151Mc RT may contribute to the observed changes in binding and incorporation of TFV-DP. The novel pattern of TFV-resistance may help adjust therapeutic strategies for NRTI-experienced patients with multi-drug resistant (MDR) mutations.

## Introduction

Nucleos(t)ide reverse transcriptase inhibitors (NRTIs) are used in combination with other classes of drugs for the treatment of patients infected with human immunodeficiency virus type-1 (HIV-1). This approach is known as highly active anti-retroviral therapy (HAART) and has been remarkably successful in reducing the viral loads and increasing the number of CD4+ cells in patients' plasma. However, prolonged therapies inevitably result in resistance to all of the available drugs. Several mutations in the reverse transcriptase (RT) are known to cause resistance to NRTIs through two basic mechanisms:

1. The excision mechanism, which is based on an enhanced capacity of RT to use adenosine triphosphate (ATP) as a nucleophile for the removal of the chain-terminating nucleotide from the DNA terminus. The excision reaction products are a 5′, 5′-dinucleoside tetraphosphate and an unblocked primer with a free 3′-OH, allowing DNA synthesis to resume [1], [2], [3]. Increased excision of NRTIs is imparted by Excision Enhancement Mutations, typically M41L, D67N, K70R, T215Y/F, L210W, and K219E/Q (also known as Thymidine Associated Mutations, or TAMs). Other mutations have also been reported to enhance excision, including insertions or deletions at the tip of the β3- β4 loop of the fingers subdomain in the background of other excision enhancement mutations [4], [5], [6], [7], [8], [9], [10], [11].
2. The other mechanism of NRTI resistance is the exclusion mechanism, which is caused when NRTI-resistance mutations in RT enhance discrimination and reduce incorporation of the NRTI-triphosphate (NRTI-TP). This mechanism is exemplified by the resistance of the M184V RT mutant to lamivudine (3TC) and emtricitabine (FTC) due to steric clash between the β-branched Val or Ile at position 184 and the oxathiolane ring of the inhibitors [12], [13]. Another example of the exclusion mechanism is the multi-drug resistant (MDR) HIV-1 RT known as Q151M complex (Q151Mc). This RT contains the Q151M mutation together with a cluster of four additional mutations (A62V/V75I/F77L/F116Y) [14], [15]. Q151M by itself causes intermediate- to high-level resistance to zidovudine (AZT), didanosine (ddI), zalcitabine (ddC), stavudine (d4T), and low level resistance to abacavir (ABC) [15], [16], [17] without reducing viral fitness [18], [19]. Addition of the four associated mutations increases replication capacity of RT and results in high-level resistance to AZT, ddI, ddC, and d4T, 5-fold resistance to ABC and low-level resistance to lamivudine (3TC) and emtricitabine (FTC) [17], [18], [19], [20], [21]. Miller *et al.* and Smith *et al.* reported a 1.8-fold and 3.6-fold increase in resistance to tenofovir (TFV), respectively [22], [23].

Biochemical studies on the mechanism of Q151Mc resistance to multiple NRTIs have revealed that the mutations of this complex decrease the maximum rate of NRTI-TP incorporation without significantly affecting the incorporation of the natural nucleotides [21], [24], [25]. Structurally, the Q151 residue interacts with the 3′-OH of a normal deoxynucleoside triphosphate (dNTP) substrate [26]. It appears that the Q151Mc mutations cause resistance to multiple NRTIs by affecting the hydrogen bond network involving protein side chains in the vicinity of the dNTP-binding site and the NRTI triphosphate lacking a 3′-OH [25], [26], [27]. The Q151Mc set of mutations was also reported to decrease pyrophosphate PPi- and ATP-mediated excision [25].

K65R is another mutation near the polymerase active site that confers NRTI resistance through the exclusion mechanism. Specifically, K65R RT has reduced susceptibility to the acyclic nucleotide analog, TFV and other NRTIs, including ddI, ddC, ABC, FTC and 3TC [28], [29], [30], [31]. Biochemical studies with K65R RT have demonstrated that this enzyme decreases the incorporation rate of these NRTIs [32], [33], [34]. The crystal structure of K65R RT in complex with DNA and TFV diphosphate (TFV-DP) revealed that R65 forms a molecular platform with the conserved residue R72, and the platform enhances the ability of K65R RT to discriminate NRTIs from dNTPs [35]. HIV carrying the Q151Mc mutations has been reported to be susceptible to TFV disoproxil fumarate (TFV-DF), the oral prodrug of TFV that enhances its oral bioavailability and anti-HIV activity [22], [36]. While the K65R mutation appeared in several patients treated for more than 18 months with TFV-DF, no patient developed multi-NRTI resistance through appearance of Q151Mc [37].

Here we report the identification of unique HIV clinical isolates that have acquired the K70Q mutation in the background of Q151Mc during TFV-DF-containing therapy. We have used a combination of virological, biochemical, and molecular modeling methods to derive the mechanism by which this mutation confers resistance to TFV.

## Materials and Methods

### Clinical samples

HIV was isolated from fresh plasma immediately after collection of clinical samples from study participants at the outpatient clinic of the AIDS Clinical Center (ACC), International Medical Center of Japan. The Institutional Review Board approved this study (IMCJ-H13-80) and a written consent was obtained from all participants.

### Construction of recombinant clones of HIV-1

Recombinant infectious clones of HIV-1 carrying various mutations were prepared using standard site-directed mutagenesis protocols as described previously [38]. The NL4-3-based molecular clone was constructed by replacing the *pol*-coding region with the HIV-1 BH10 strain. Restriction enzyme sites *Xma I* and *Nhe I* were introduced by silent mutations into the molecular clone at positions corresponding to HIV-1 RT codons 15 and 267, respectively [39]. Each molecular clone was transfected into COS-7 cells. Cells were grown for 48 h, and culture supernatants were harvested and stored at −80°C until use.

### Single-cycle drug susceptibility assay

Susceptibilities to various RT inhibitors were determined using the MAGIC-5 cells which are HeLa cells stably transfected with a β-galactosidase gene under the control of an HIV long terminal repeat promoter, and with vectors that express the CD4 receptor and the CCR5 co-receptor under the control of the CMV promoter as described previously [40]. Briefly, MAGIC-5 cells were infected with diluted virus stock (100 blue forming units) in the presence of increasing concentrations of RT inhibitors, cultured for 48 h, fixed, and stained with X-Gal (5-bromo-4-chloro-3-indolyl-β-D-galacto-pyranoside). The stained cells were counted under a light microscope. Drug concentrations reducing the number of infected cells to 50% of the drug-free control (EC_50_) were determined from dose response curves.

### Enzymes

RT sequences coding for the p66 and p51 subunits of BH10 were cloned in the pRT dual vector, which is derived from pCDF-2 with LIC duet minimal adaptor (Novagen), using restriction sites *PpuMI* and *SacI* for the p51 subunit, and *SacII* and *AvrII* for the p66 subunit. RT was expressed in the *Escherichia coli* strain BL21 (Invitrogen) and purified by nickel affinity chromatography and MonoQ anion exchange chromatography [41]. RT concentrations were determined spectrophotometrically based on absorption at 260 nm using a calculated extinction coefficient (261,610 M^−1^ cm^−1^). The active site concentration of the various RT preparations was calculated as described below.

### Nucleic acid substrates

DNA oligomers were synthesized by Integrated DNA Technologies (Coralville, IA). An 18-nucleotide DNA primer fluorescently labeled with Cy3 at the 5′ end (P_18_; 5′-Cy3 GTC CCT GTT CGG GCG CCA-3′) and a 100-nucleotide DNA template (T_100_; 5′-TAG TGT GTG CCC GTC TGT TGT GTG ACT CTG GTA ACT AGA GAT CCC TCA GAC CCT TTT AGT CAG TGT GGA AAA TCT CTA GCA GTG GCG CCC GAA CAG GGA C-3′) were used in primer extension assays. An 18-nucleotide DNA primer 5′-labeled with Cy3 (P_18_; 5′-Cy3 GTC ACT GTT CGA GCA CCA-3′) and a 31-nucleotide DNA template (T_31_; 5′- CCA TAG CTA GCA TTG GTG CTC GAA CAG TGA C-3′) were used in the ATP rescue assay and pre-steady state kinetic experiments.

### Active site titration and determination of the dissociation constant for DNA binding (K_D-DNA_)

Determination of active site concentrations in the different preparations of WT and mutant RTs were performed using pre-steady state burst experiments. A fixed concentration of RT (80 nM, determined by absorbance measurements) was pre-incubated with increasing concentrations of DNA/DNA template/primer (T_31_/P_18_), followed by rapidly mixing with a reaction mixture containing MgCl_2_ and dATP, at final concentrations of 5 mM and 50 µM, respectively. The reactions were quenched at various times (10 ms to 5 s) by adding EDTA to a final concentration of 50 mM. The amounts of product (P_18_-dAMP) were quantitated and fit to the following burst equation:
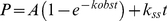
(1)where *A* is the amplitude of the burst phase that represents the RT-DNA complex at the start of the reaction, *k*_obs_ is the observed burst rate constant for dNTP incorporation, *k*_ss_ is the steady state rate constant, and *t* is the reaction time. The rate constant of the linear phase (*k_cat_*) can be estimated by dividing the slope of the linear phase by the enzyme concentration. The active site concentration and template/primer binding affinity (K_D-DNA_) were determined by plotting the amplitude (*A*) against the concentration of template/primer. The data were fit using non-linear regression to a quadratic equation:
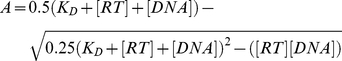
(2)where *K_D_* is the dissociation constant for the RT-DNA complex, and [RT] is the concentration of active polymerase molecules. Subsequent biochemical experiments were performed using corrected active site concentrations [42], [43].

### Primer extension assay

To examine the DNA polymerase activity of WT and mutant RTs and the inhibition of DNA synthesis by TFV, the primer extension assays were carried out on the T_100_/P_18_ template/primer (P_18_ was 5′-Cy3 labeled) in the presence or absence of 3.5 mM ATP [41]. The enzyme (20 nM active sites) was incubated with 20 nM template/primer at 37°C in a buffer containing 50 mM Tris-HCl, pH 7.8 and 50 mM NaCl. The DNA synthesis was initiated by the addition of 1 µM dNTP and 10 mM MgCl_2_. The primer extension assays were carried out in the presence or absence of varying concentrations of TFV-DP. The reactions were terminated after 15 min by adding equal volume of 100% formamide containing traces of bromophenol blue. The extension products were resolved on a 7 M urea-15% polyacrylamide gel, and visualized by phosphor-imaging (FLA 5100, Fujifilm, Tokyo). We followed standard protocols that utilize the Multi Gauge software (Fujifilm) to quantitate primer extension [41], [44]. The results from dose response experiments were plotted using Prism 4 (GraphPad Software Inc., CA) and IC_50_ values for TFV-DP were obtained at midpoint concentrations.

### ATP-dependent rescue assay

Template/primer (T_31_/P_18_) terminated with TFV (T/P_TFV_) was prepared as described in Michailidis et al [41]. 20 nM of T/P_TFV_ was incubated at 37°C with HIV-1 RT (60 nM), either at various concentrations of ATP (0–7 mM) for 30 minutes, or for various times (0–120 minutes) with 3.5 mM ATP, in RT buffer containing 50 mM Tris-HCl, pH 7.8, and 50 mM NaCl, and 10 mM MgCl_2_. The assay was performed in the presence of excess competing dATP (100 µM) that prevented reincorporation of the excised TFV, 0.5 µM dTTP and 10 µM ddGTP. Reactions were quenched with 100% formamide containing traces of bromophenol blue and analyzed as described above. The dissociation constants (K_d_) of the various enzymes for ATP used in the rescue reactions were determined by fitting the rescue data at various ATP concentrations, using non-linear regression fitting to hyperbola.

### Kinetics of dNTP incorporation by WT and mutant enzymes

To determine the binding affinity of WT and mutant enzymes to the dNTP substrate (K_D-dNTP_) and to estimate the maximum rate of dNTP incorporation by these enzymes (*k_pol_*), we carried out transient-state experiments using a rapid quench instrument (RQF-3, Kintek Corporation, Clarence, PA) at 37°C in RT buffer (50 mM Tris-HCl, pH 7.8 and 50 mM NaCl). HIV-1 RT (50 nM active sites) was pre-incubated with 50 nM T_31_/P_18_ in one syringe (Syringe A), whereas varying concentrations of dNTP and 10 mM MgCl_2_ were kept in another syringe (Syringe B). The solutions were rapidly mixed to initiate reactions, which were subsequently quenched at various times (5 ms to 10 s) by adding EDTA to a final concentration of 50 mM. The products from each quenched reaction were resolved, quantitated, and plotted as described above. The data were fit by non-linear regression to the burst equation (Eq 1).

To obtain the dissociation constant K_D-dNTP_ for dNTP binding to the RT-DNA complex, the observed burst rates (*k_obs_*) were fit to the hyperbolic equation (Eq. 3) using nonlinear regression:
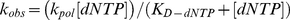
(3)where *k_pol_* is the optimal rate of dNTP incorporation.

The kinetics of TFV incorporation by the WT and mutant enzymes were carried out in a manner similar to that employed for natural dNTP substrate except the time of reactions. It was noted that the mutant enzymes required longer time to incorporate TFV compared to the WT HIV-1 RT (detailed in the Results section).

### Molecular Modeling

Molecular models of mutant enzymes were generated using SYBYL (Tripos Associates, St. Louis, MO). The starting protein coordinates were from the crystal structure of HIV-1 RT in complex with DNA template/primer and TFV-DP (PDB file 1T05) [45]. They were initially modified by the Protein Preparation tool (Schrodinger Molecular Modeling Suit, NY), which deletes unwanted water molecules, sets charges and atom type of metal ions, corrects misoriented Gln and Asn residues, and optimizes H-atom orientations. Amino acid side chains were substituted in by Maestro (Schrodinger, Molecular Modeling Suite, NY). Molecular dynamics simulations of the WT and mutant RT models were carried out to obtain the most stable structures by Impact, interfaced with Maestro at constant temperature, and OPLS_2005 force field. The molecular dynamics simulations were performed for 1000 steps with 0.001 ps intervals. The temperature relaxation time was 0.01 ps. The Verlet integration algorithm was used in simulations. The structures were imported into Pymol (http://www.pymol.org) for visualization and comparison.

## Results

### Phenotypic resistance to TFV-DF in the absence of any known TFV resistance mutations

**Figure 1:**
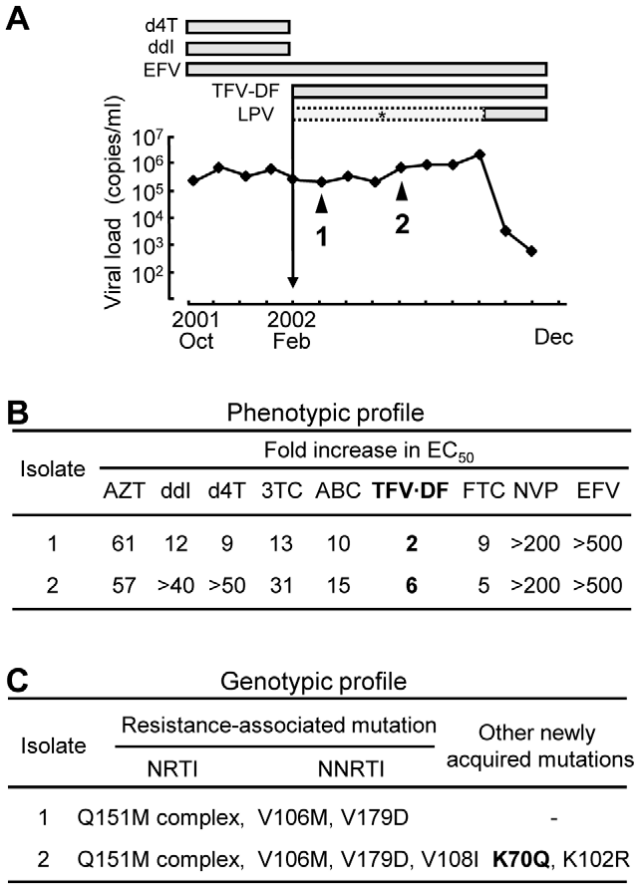
Clinical course of patient and drug resistance profile. (A) The two clinical isolates were collected from the patient at the time points indicated by triangles. Both isolates had no known resistant mutations in the protease region. During the period indicated by asterisk, LPV was administrated but the patient demonstrated poor adherence due to undesirable side effects. After instruction on the use of antiretroviral drugs, the viral loads successfully decreased below the detection limit (<50 copies/ml). (B) Phenotypic drug susceptibility assays of clinical isolates in at least three independent experiments are shown as a relative increase in EC_50_ compared to HIV-1 NL4-3 strain which served as WT (see also Table S1). (C) Mutations observed in the isolates that are defined as the NRTI and NNRTI resistance associated mutations deposited in the HIV Drug Resistance Database maintained by International AIDS Society 2009 [58] and the Stanford University (http://hivdb.stanford.edu/) were shown. Abbreviations of drugs used: d4T, stavudine; ddI, didenosine; EFV, efavirenz; TFV-DF, tenofovir disoproxil fumarate; LPV, lopinovir; AZT, zidovudine; 3TC, lamivudine; ABC, abacavir; FTC, emtricitabine; NVP, nevirapine.

During phenotypic and genotypic evaluation of the clinical isolates we identified a unique virus that exhibited an apparent discordance between the phenotypic and genotypic results. The clinical history of the patient and the corresponding genotypic and phenotypic changes during the course of the therapy are summarized in Fig. 1. (Also see Table S1). The patient's treatment before Feb 2002 included d4T, ddI, and EFV and did not decrease significantly the viral loads (Fig 1A). Hence, the therapeutic regimen was switched to TFV-DF, EFV, and the protease inhibitor lopinavir (LPV). However, the patient's immunological and virological responses still did not improve due to poor adherence, especially to LPV. Genotypic and phenotypic analyses on March 2002 (point 1) and June 2002 (point 2) revealed resistance to multiple RT inhibitors, including NNRTIs (Fig 1B). Resistance to all NRTIs, except AZT and FTC, was enhanced in the point 2 isolate (Fig. 1B). Notably, this isolate showed an increase in resistance to TFV-DF in the absence of the canonical TFV resistance mutation (K65R) and in the presence of Q151Mc mutations (Fig. 1C). Previously, it has been shown that Q151Mc remains susceptible to TFV [22] although Smith *et al.* reported that Q151Mc had a 3.6-fold increase in TFV resistance [23]. Suppression of the viral load was finally achieved by improvement in drug adherence to LPV and by the addition of FTC in the therapeutic regimen, since no protease resistance mutations were found within the protease coding region.

To identify the mutation(s) responsible for the unexpected resistance to TFV-DF we sequenced the entire RT coding region at time-points 1 and 2 (Figure S1, GenBank Accession Number AB506802 and AB506803). Of the three substituted residues (70, 102, and 108) amino acids 102 and 108 are part of the structurally distinct NNRTI binding pocket [46], which can mutate during EFV-based therapeutic regimens. However, residue 70 is located in the β3-β4 hairpin loop of the p66 “fingers” subdomain of HIV-1 RT, which interacts with the incoming dNTP substrate [10], [27]. Different mutations at this site have been previously implicated in NRTI resistance [47], suggesting that the observed K70Q mutation may be involved in the increased resistance to TFV-DF.

### NRTI resistance enhancement by mutation at residue 70

Several mutations at position 70 of HIV-1 RT (R, G, E, T, N and Q) have been reported to the Stanford HIV-1 Drug Resistance Database (http://hivdb.stanford.edu/, accessed on Feb. 27^th^ 2010). K70Q is rarely observed in treatment-naïve patients (0.04%), but appears more often in clinical samples from NRTI-treated patients (0.1%, p<0.0001 compared with the frequency of K70Q in treatment-naïve patients) but not NNRTI-treated patients. Furthermore, K70Q is observed in 0.5% of the clinical samples from patients infected with HIV-1 Q151M. There have been no previous reports on a possible role of K70Q in NRTI resistance.

**Figure 2:**
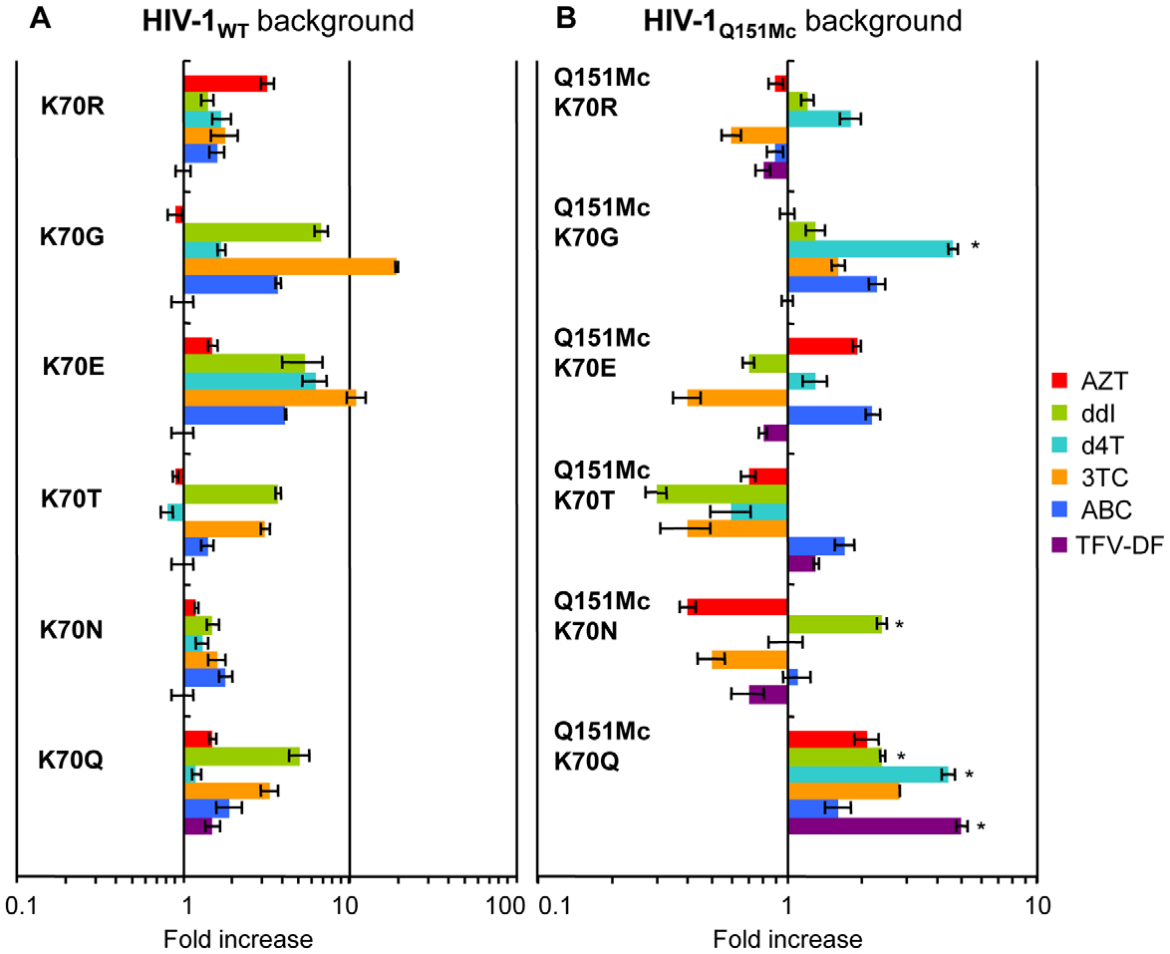
NRTI resistance of HIVs with mutations at RT residue 70 in the background of WT or Q151Mc. Antiviral activities of HIV-1s carrying mutations at residue 70 (K70R, K70G, K70E, K70T, K70N, or K70Q) in the WT (A) or Q151Mc (B) background were determined by the MAGIC5 assay. The data for each clone were compared to WT (A) and Q151Mc (B) HIV-1 and are shown as fold increase; AZT (red), ddI (green), d4T (cyan), 3TC (orange), ABC (blue), and TFV-DF (purple). Error bars represent standard deviations from at least three independent experiments (see also Table S2 and Table S3). The asterisk indicates statistically significant in EC_50_ values (*P*<0.0001 by t-test).

To examine the effect of K70Q on drug susceptibility we generated a series of HIV variants with mutations at RT codon 70 (Figure 2A and also Table S2). The HIV-1_K70Q_ variant exhibited marginal resistance to ddI and 3TC (5- and 3.3-fold, respectively), but no significant resistance to other NRTIs. We further examined whether the mutations at residue 70 affect susceptibility to NRTIs in the Q151Mc background (Figure 2B and also Table S3). HIV-1_K70G/Q151Mc_ had enhanced resistance to d4T (4.6-fold) as compared to HIV-1_Q151Mc_. Notably, HIV-1_K70Q/Q151Mc_ also showed enhanced resistance to ddI and d4T (2.4- and 4.4-fold, respectively, compared to HIV-1_Q151Mc_). In addition, HIV-1_K70Q/Q151Mc_ displayed 5-fold increased resistance to TFV-DF compared to HIV-1_Q151Mc_. Other K70 mutations exhibited little or no resistance to TFV-DF.

### Primer Extension and ATP-based Rescue Assays

**Figure 3:**
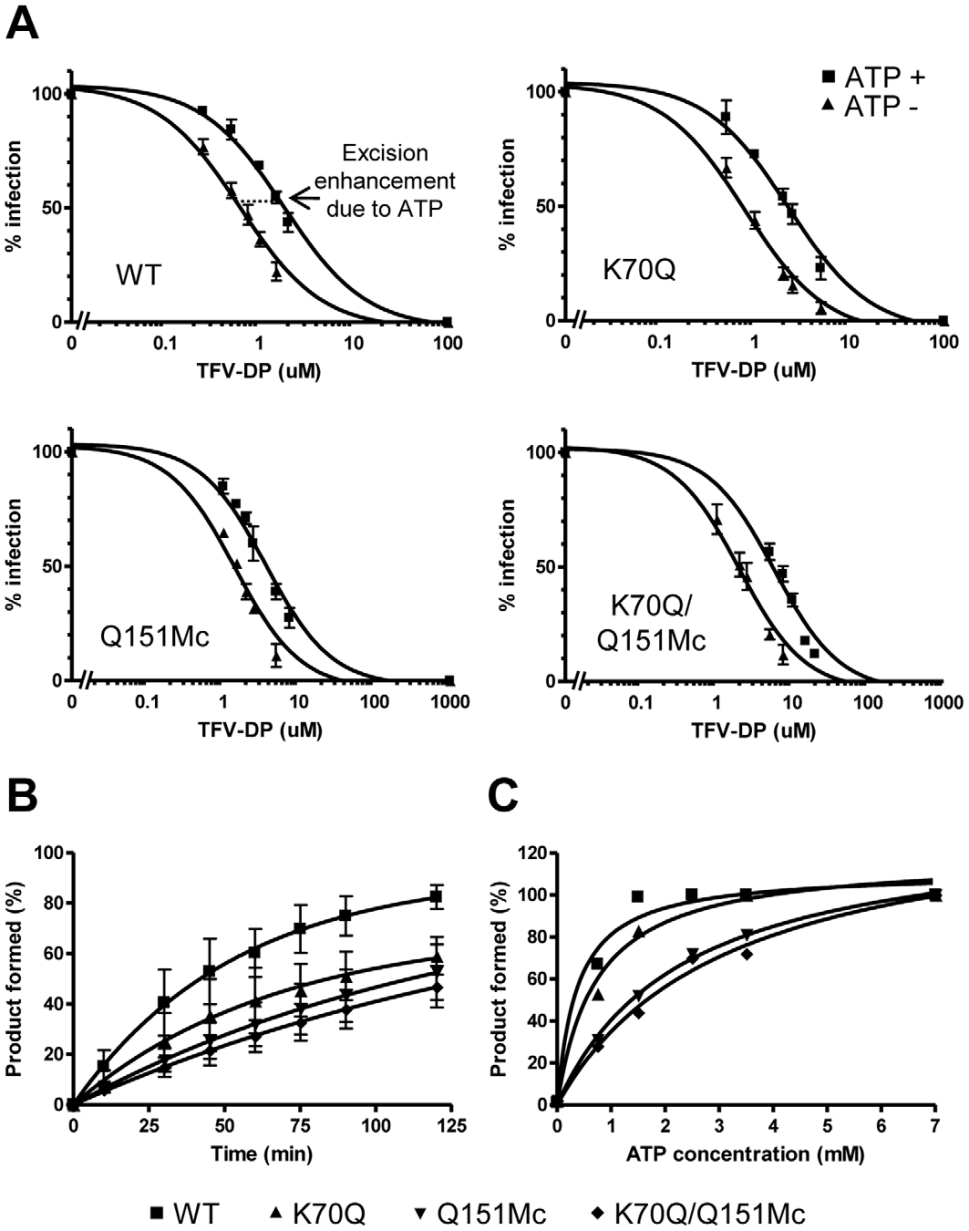
Effects of RT mutations K70Q, Q151Mc, or K70Q/Q151Mc on DNA primer extension activity and on ATP-based excision activities. (A) Effect of varying concentrations of TFV-DP on the primer extension activities of HIV-1 WT and mutant RTs. The experiments were carried out in the presence (▪) or absence (▴) of 3.5 mM ATP (B) Time dependence of ATP-based rescue of TFV-terminated primers. TFV-terminated T_31_/P_18_ oligos (20 nM) were incubated with 60 nM RT and 3.5 mM ATP. The reaction mixture also included excess of competing dATP (100 µM) that prevented reincorporation of TFV-DP and 0.5 µM dTTP, and 10 µM ddGTP that allowed extension of the rescued primer by two nucleotides and chain termination. Rescue products (WT [▪], K70Q [▴], Q151Mc [▾] and K70Q/Q151Mc [♦]) were analyzed at indicated time points. (C) ATP-based rescue was dependent on concentration of ATP. Reactions were as in (B), but for 30 minutes and at varying concentrations of ATP. Rescue products at 7 mM ATP are defined as 100% product formed.

As mentioned earlier, a key mechanism of NRTI resistance is the excision mechanism, which is based on the enhanced ability of NRTI-resistant enzymes to use ATP for unblocking chain-terminated primers and allow for further DNA synthesis to continue [2], [3], [48]. To determine whether the K70Q mutation causes TFV resistance through the excision mechanism we measured the susceptibility of WT and mutant RTs to inhibition by TFV in the presence or absence of ATP. In gel-based assays, an enhancement in excision would manifest as an increase in the production of fully extended DNA when 3.5 mM ATP is included in the extension reaction [49], [50]. Our extension assays in the absence of ATP (no-excision conditions) showed that addition of the K70Q mutation to Q151Mc HIV-1 RT enhances resistance to TFV-DP. However, this enhancement is not influenced by the presence of ATP (Table 1, Fig. 3A and Figure S2A). In fact, excision enhancement due to the presence of ATP measured as [IC_50_ with ATP]/[IC_50_ without ATP] was similar for all enzymes, including the WT RT (from 2.7-fold to 2.9-fold for WT, K70Q, Q151Mc, and K70Q/Q151Mc RTs) (Table 1). Using a related type of assay, the ATP-mediated rescue assay, we compared the rates by which the WT and mutant RTs unblock TFV-terminated primers and extend products past the point of chain-termination. We find that the ATP-based rescue activity of WT RT is not slower, but 1.5-, 2.5-, and 3-fold faster than that of K70Q, Q151Mc, and K70Q/Q151Mc RTs, respectively (Fig. 3B and Figure S2B). In addition, the ATP-based rescue activity of WT RT was saturated at lower concentrations of ATP than K70Q, Q151Mc, and K70Q/Q151Mc RTs (the apparent K_D-ATP_ for WT, K70Q, Q151Mc and K70Q/Q151Mc were 0.4, 0.7, 2.3, and 3.1 mM, respectively), suggesting that a better binding of ATP may contribute to the slightly enhanced excision activity of WT RT (Fig. 3C and Figure S2C). Collectively, these results rule out the possibility that K70Q/Q151Mc becomes resistant to TFV through the excision mechanism.

**Table 1:** Primer extension assay in the presence or absence of ATP.

Enzyme^a^	IC_50_ (nM) of TFV-DP^b^ (fold increase^c^)		Excision enhancement due to ATP^d^
	Without ATP	With ATP	
WT	641±83	1854±197	2.9
	(1)^b^	(1)^b^	
K70Q	802±99	2306±270	2.9
	(1.3)	(1.2)	
Q151Mc	1503±90	3996±341	2.7
	(2.3)	(2.1)	
K70Q/Q151Mc	2392±353	7001±226	2.9
	**(3.7)**	**(3.8)**	

a. The sequence of HIV RT WT and mutant derived from BH10.

b. Data are means ± standard deviations from at least three independent experiments.

c. The relative increase in IC_50_ value compared with each HIV-1 RT WT without, or with ATP is given in parentheses. Bold indicates an increase in fold increase value greater than 3-fold.

d. Excision enhancement due to ATP is calculated as IC_50_ with ATP/IC_50_ without ATP.

### Pre-Steady Kinetic Constants for Binding and Incorporation of dATP and TFV-DP

**Figure 4:**
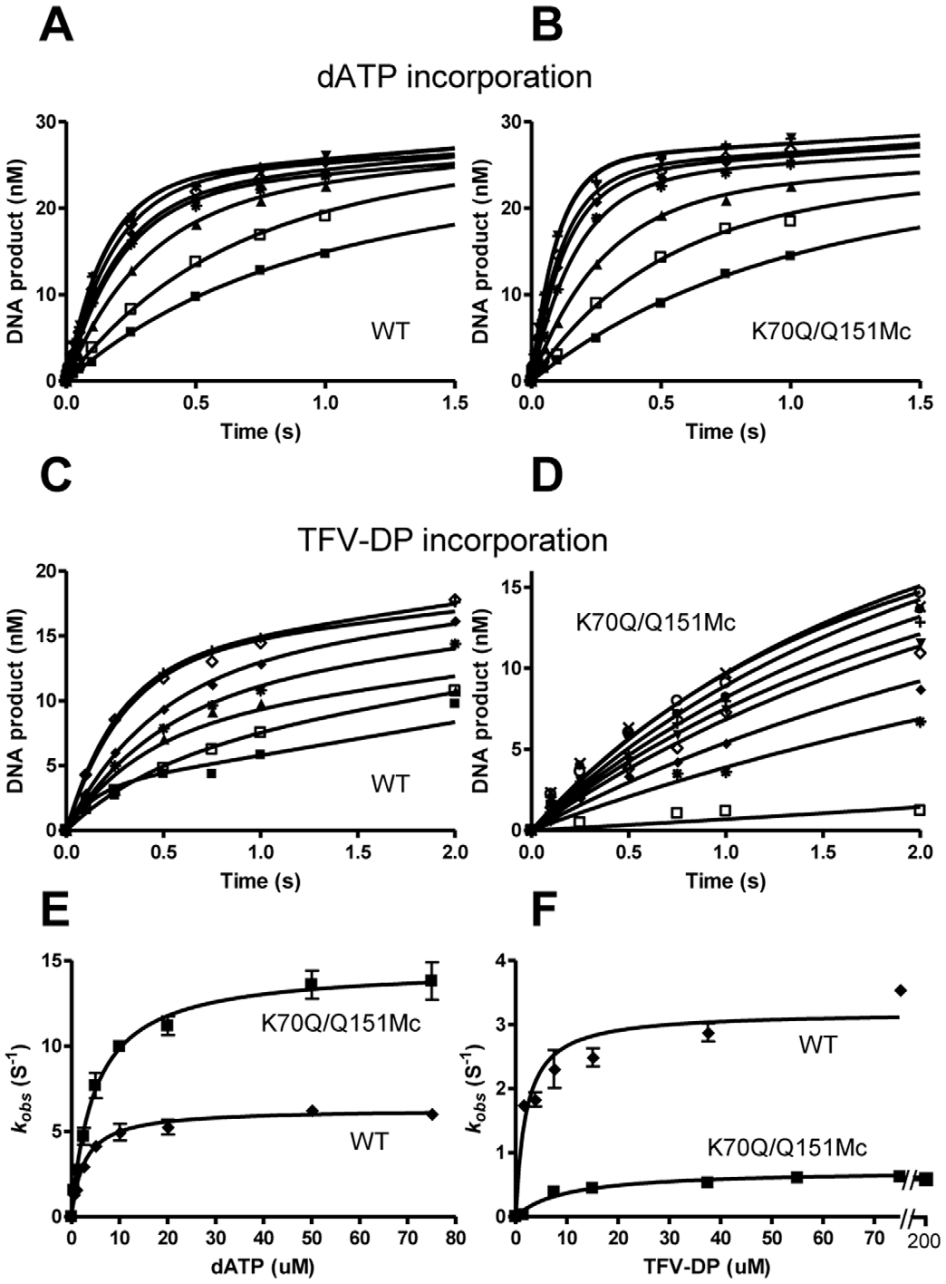
Pre-steady state kinetics of incorporation of dATP or TFV-DP by WT and K70Q/Q151Mc HIV-1 RTs. Single-nucleotide incorporation of dATP (panels A, B, and E) or TFV-DP (panels C, D, and F) by WT (panels A, C, E, and F) and K70Q/Q151Mc (panels B, D, E, and F). Formation of extended primer products in the reactions with WT RT and K70Q/Q151Mc RT were measured at 5 ms to 5 s time points, using the following dATP concentrations: 0.5 (▪), 1 (□), 2.5 (▴), 5 (*), 10 (♦), 20 (◊), 50 (▾) and 75 µM (+). Incorporation of TFV was measured at 0.1–10 s reactions and at the following TFV-DP concentrations: 0.75 (▪), 1.5 (□), 3.75 (▴), 7.5 (*), 15 (♦), 37.5 (◊) and 75 µM (+) for reactions with WT RT (panel C), and 1.5 (□), 7.5 (*), 15 (♦), 37.5 (◊), 55 (▾), 75 (+), 112.5 (•), 150 (○) and 200 µM (x) for reactions with K70Q/Q151Mc RT (panel D). (E) The amplitudes of the burst phases from the dATP reactions shown in panels A (WT, [♦]) and B (K70Q/Q151Mc, [▪]) were plotted as a function of dATP concentrations. (F) The amplitudes of the burst phases from the TFV-DP reactions shown in panels C (WT, [♦]) and D (K70Q/Q151Mc, [▪]) were plotted as a function of TFV-DP concentrations. The solid lines in panels A, B, C, and D represent the best fit of data to the burst equation. Each point represents the average values of three experiments.

To determine whether the resistance by K70Q/Q151Mc is caused by an increased preference of physiological dATP substrate over TFV-DP, we carried out pre-steady state transient kinetic analyses of WT, K70Q, Q151Mc, and K70Q/Q151Mc enzymes. The kinetic constants *k_pol-dATP_* and K_D-dATP_ for WT and mutant enzymes are presented in Table 2 and Fig. 4 (and also in Figure S3). The results reveal that K70Q, Q151Mc, and K70Q/Q151Mc RTs have increased *k_pol-dATP_* as well as K_D-dATP_. Both Q151Mc and K70Q/Q151Mc enzymes incorporate dATP faster than WT (17.9 and 14.6 s^−1^, respectively *vs.* 6.3 s^−1^) but have a weaker binding affinity for dATP than WT RT (5.4 and 5.0 µM, respectively *vs.* 2.6 µM). Hence, the catalytic efficiency ratio of dATP incorporation remains similar for all enzymes (*k_pol-dATP_*/K_D-dATP_ ratios for WT, K70Q, Q151Mc, and K70Q/Q151Mc were 2.4, 2.2, 3.3, and 2.9 µM^−1^·s^−1^, respectively). On the contrary, a significant change in the incorporation efficiency of TFV was observed. The K70Q and K70Q/Q151Mc enzymes had more than 4.5-fold reduced affinity for TFV than the WT enzyme (K_D-TFV_ values were 8.6 and 8.9 µM compared to 1.9 µM). In addition, the turnover rates of TFV incorporation by the WT and K70Q enzymes were comparable (*k_pol-TFV_* were 2.8 and 3.1 s^−1^, respectively). The addition of the K70Q mutation to Q151Mc also reduced the *k_pol_* for TFV-DP. The net effect of these changes was a significant reduction in the TFV-DP incorporation efficiencies of the mutant enzymes compared to the WT enzyme (*k_pol-TFV_*/K_D-TFV_ ratios for WT, K70Q, Q151Mc, and K70Q/Q151Mc were 1.47, 0.36, 0.3, and 0.11 µM^−1^·s^−1^, respectively; Table 2). WT RT incorporated TFV-DP most efficiently, followed by K70Q>Q151Mc>K70Q/Q151Mc enzymes. As a direct measure of the enzyme's ability to discriminate between the natural dATP substrate and the TFV, we determined the “selectivity”, defined as the ratio of efficiency of the enzyme to incorporate dATP over TFV-DP (*k_pol-dATP_*/K_D-dATP_/*k_pol-TFV_*/K_D-TFV_). The selectivity values demonstrate that the K70Q/Q151Mc enzyme favors incorporation of dNTP over TFV-DP 26.3 times compared to 1.6 times by the WT enzyme, leading to a 16.4-fold resistance to TFV (defined as selectivity_mutant_/selectivity_WT_; Table 2). This resistance is more than twice the TFV resistance of Q151Mc and 4 times the TFV resistance of K70Q.

**Table 2:** Pre-steady state kinetic constants for binding and incorporation of dATP and TFV-DP by WT, K70Q, Q151Mc and K70Q/Q151Mc HIV-1 RT.

Pre-steady state kinetic constants^a^								
Enzyme^b^	dATP			TFV-DP			Selectivity^c^	Resistance^d^
	*k*_pol_ (s^−1^)	K_d_ (µM)	*k*_pol_/K_d_ (µM^−1^·s^−1^)	*k*_pol_ (s^−1^)	K_d_ (µM)	*k*_pol_/K_d_ (µM^−1^·s^−1^)		
WT	6.3±0.5	2.6±0.1	2.4±0.2	2.8±0.08	1.9±0.2	1.47±0.07	1.6	-
K70Q	8.4±0.4	3.8±0.6	2.2±0.4	3.1±0.4	8.6±1.5	0.36±0.08	6.1	3.8
Q151Mc	17.9±0.4	5.4±0.5	3.3±0.3	1.3±0.03	4.3±0.8	0.3±0.06	11	6.9
K70Q/Q151Mc	14.6±1.6	5.0±0.07	2.9±0.3	1.0±0.03	8.9±2.1	0.11±0.03	26.3	16.4

a. Data are means ± standard deviations from at least three independent experiments.

b. The sequence of HIV RT WT and mutant derived from BH10.

c. Selectivity is defined as (*k*_pol_/K_d_)_dATP_/(*k*_pol_/K_d_)_TFV-DP_.

d. Resistance (fold) is calculated as selectivity _mutant_/selectivity _WT_.

### Molecular modeling

**Figure 5:**
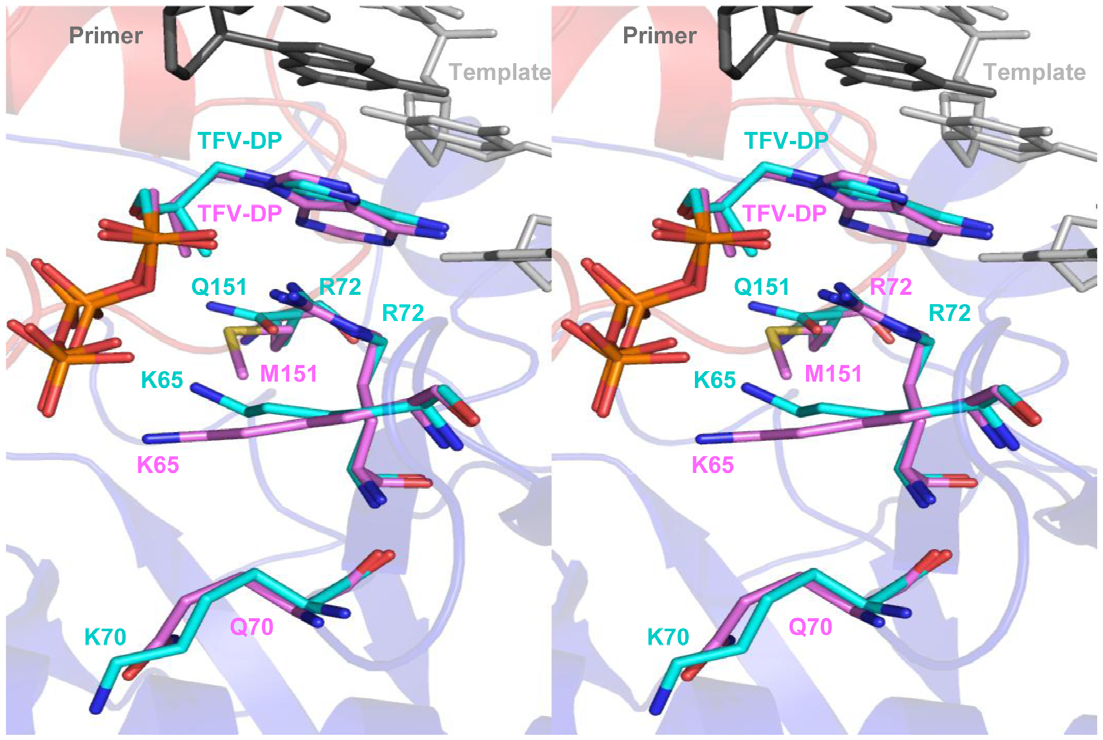
Stereo view of TFV-DP in the polymerase active site of WT RT and K70Q/Q151Mc RT. WT RT residues are shown as cyan sticks, K70Q/Q151Mc RT residues are shown as purple sticks. The primer strand is shown as dark gray sticks, template strand as light gray sticks. The fingers and palm subdomains are shown as blue and red cartoons, respectively.

Molecular dynamics simulations on the control structural coordinates of the WT RT/DNA/TFV-DP crystal structure [45] did not cause any significant structural changes, suggesting that the modeling protocols do not alter the structures in ways that are not related to the K70Q or Q151Mc mutations. The root mean square deviation (rmsd) between the Cα atoms of the WT structures before and after simulation was 0.1 Å. Similarly, the rmsd between the Cα atoms of WT and mutant RT molecular models were also very low (∼0.1 Å). Comparison of these models showed a significant repositioning of residue 65 in Q151Mc/K70Q (Fig. 5), and to a lesser extent in K70Q or Q151Mc RTs (not shown). Additional smaller changes in the side chains of residues 151, 70, and 72 were also observed (Fig. 5). The structure of TFV-DP was also slightly adjusted, possibly as a result of the changes in the surrounding residues (Fig. 5). While residue 70 is located proximal to residue 65, and to the phosphates of the incoming TFV-DP, it does not appear to interact directly with these structural elements.

## Discussion

We have discovered a novel HIV mutation that causes high-level resistance to TFV-DF. We have also determined the biochemical mechanism of this resistance. TFV-DF is a valuable NRTI therapeutic option for patients infected with multi-drug resistant Q151Mc HIV-1 [22]. We demonstrate here that Q151Mc can acquire an additional mutation, K70Q, which expands the multi-drug resistance to include high-level resistance to TFV-DF. We identified this mutant during genotypic analysis of clinical isolates from an HIV-infected patient who was not responding to TFV-DF. The K70Q/Q151Mc set of mutations is currently rare among HIV-infected patients. However, we believe that similar to K65R, its prevalence will increase, as tenofovir use continues to rise. Our virological studies with recombinant viruses confirmed that the observed enhancement and expansion of multi-drug resistance is the consequence of the addition of K70Q to Q151Mc HIV. Recently, the concept of clinical cut-offs (CCOs) has been introduced to improve the prediction of drug resistance during antiretroviral therapies. CCOs are better correlated with virologic response than biological cut-offs [51], [52]. Importantly, K70Q/Q151Mc is 10 times less susceptible to TFV-DF than WT HIV-1, whereas the CCOs for TFV-DF is defined as a 2.1-fold reduction in virologic response to this inhibitor. Moreover, K70Q/Q151Mc is at least twice as resistant to TFV as the well-known TFV-resistant K65R in the background of Q151Mc (as reported in the Stanford HIV Drug Resistance Database).

Previous studies have offered insights into the drug resistance mechanism of similar mutations (K70E, K70G, K70R, and K70T). Specifically, K70E was selected in patients with virological failure after TFV-DF-based antiviral therapy [53], [54], [55]. K70T emerged in the background of Q151Mc during *in vitro* selection by TFV-DF [56]. K70R is a key mutation involved in resistance to AZT and appears in the background of other excision enhancement mutations [2], [3], [57]. In our case, a new mutation (K70Q) was identified in a patient infected with Q151Mc HIV-1 during the course of TFV-DF-based antiviral therapy. The International AIDS Society-USA publishes [58] every year a list of HIV-1 drug resistance mutations compiled by a panel of experts charged with the goal of delivering accurate, unbiased, and evidence-based information for use by HIV clinical practitioners. In order for a novel mutation to be accepted in the list it should meet at least *one* of the following criteria: 1) *in vitro* passage experiments or validation of contribution to resistance by using site-directed mutagenesis; 2) susceptibility testing of laboratory or clinical isolates; 3) nucleotide sequencing of viruses from patients in whom the drug is failing; 4) correlation studies between genotype at baseline and virologic response in patients exposed to a drug. Our study has unambiguously demonstrated that K70Q meets at least the first three criteria: evidence for criterion #1 is shown in Figure 2; for criterion #2 in Figures 1 and 2; and for criterion #3 in Figure 1 and Figure S1. Therefore, the K70Q mutation meets the criteria of a clinically relevant mutation.

In addition to the clinical and virological studies, we used biochemical techniques to determine the mechanism of TFV resistance imparted by the K70Q mutation to Q151Mc RTs. We used primer extension assays to show that K70Q/Q151Mc RT is less susceptible to TFV-DP than WT and Q151Mc RTs. We demonstrated that the mechanism of this resistance is not based on excision. On the contrary, we showed that the ATP-based excision of the mutant enzymes was slightly decreased with respect to WT RT, possibly because of decreased affinity of the mutant enzymes for the ATP excision substrate, incurred by changes in the binding environment of ATP, such as the loss of lysine at position 70.

Using transient-state kinetics we unambiguously established that the overall mechanism of K70Q/Q151Mc resistance to TFV is due to enhanced discrimination between the natural dATP substrate and TFV-DP. While all mutant enzymes had comparable efficiency of dATP incorporation, they displayed varying affinity and turnover rates of incorporation. It appears that the stronger effect of the enhanced discrimination overcomes the slight increase in sensitivity due to the small increase in excision. As a result, the mutant enzymes are resistant to the inhibitor.

Mutations at position 70 of RT have been known to confer NRTI resistance by two distinct mechanisms: K70R combined with at least two excision enhancing mutations, D67N and T215Y, enhances ATP-mediated excision of AZT and d4T [1], [2], [3], [48] (*excision–dependent mechanism*). On the other hand, K70E causes resistance to 3TC, TFV, and ABC by lowering the maximum rate of inhibitor incorporation by RT (*k_pol_-dependent exclusion mechanism*) [55]. Our results establish that in the background of Q151Mc, K70Q causes TFV resistance through a third mechanism: by decreasing the binding affinity of the inhibitor (K_d_-*dependent exclusion mechanism*). Taken together, these findings highlight the remarkable ability of RT to use separate mutations at a single position to acquire NRTI resistance through three different mechanisms.

Our cell-based assays with infectious HIV-1 show that Q151Mc remains susceptible to TFV-DF, a finding consistent with previous reports [22]. Similarly, clinical isolates deposited at the Stanford HIV resistance database and carrying the Q151Mc mutation were also susceptible to TFV-DF, unless they also had the K65R mutation. However, pre-steady state characterization of TFV-DP incorporation by Q151Mc in this work (Table 2) and by others [59] showed that Q151Mc is less susceptible to TFV-DP than WT RT. This small discrepancy may be the result of potential differences in DNA-dependent and RNA-dependent DNA synthesis, or the result of the slightly increased excision of Q151Mc RT compared to WT RT (Fig. 3B and C).

To gain insights into the possible structural changes caused by the addition of K70Q to Q151Mc, we compared the molecular model of K70Q/Q151Mc RT/DNA/TFV-DP with the crystal structure of WT RT/DNA/TFV-DP [45] (Fig. 5). The network of hydrogen bonds involving the side-chains of K65, R72, and Q151 in the WT structure [26], [27], [54], is disrupted in the mutant structure. Also, Q151M and associated mutations A62V, V75I, and F77L are likely to modify the hydrophobic core of the fingers. We and others have previously shown that the side-chains of residues 72 and 65 interact with each other [35] and with Q151 and the α- and γ-phosphates of the incoming dNTP [26] or TFV-DP [45]. The functions of these residues have been established by several biochemical studies [21], [25], [60], [61], [62], [63]. The reduction in polymerase rate (*k_pol_*) and in binding affinity for TFV-DP (increased K_d.TFV-DP_) may be the consequence of one or more such structural changes. Our molecular dynamics simulation experiments suggested a re-arrangement in the position of the side chain of K65, which is a catalytically important residue. While the precise effect of this change is not clear at this point, such changes could influence the overall binding of the substrate and/or the rate of nucleotide incorporation. Moreover, such movement of K65 in the presence of a mutation at position 70 is consistent with our previously reported crystallographic data, which established that there is an interplay between the positioning of the side chains at positions 70 and 65 [64]. Ongoing crystallographic studies are expected to provide more detailed structural insights into the role of K70Q in drug resistance.

In summary, we report here clinical data showing that addition of the K70Q mutation to the Q151Mc background confers high-level HIV resistance to TFV-DF and enhances resistance to other NRTIs. The biochemical mechanism of the TFV resistance is based on reduced binding affinity and incorporation of TFV-DP. Detection of this novel pattern of TFV-DF resistance may help adjust therapeutic regimens for the treatment of patients infected with multi-drug resistant HIV-1.

## Supporting Information

Figure S1 Amino acid sequence alignment of the RT regions (amino acid 32 to 560) of the clinical isolates at time points 1 to 2 (see Figure 1A).
(DOC)

Figure S2 Effects of RT mutations K70Q, Q151Mc, or K70Q/Q151Mc on DNA primer extension activity and on ATP-based excision activities. (A) Effect of varying concentrations of TFV-DP on the primer extension activities of HIV-1 WT and mutant RTs. The experiments were carried out in the presence and absence of 3.5 mM ATP (marked as ATP (+) and ATP (−), respectively). Addition of ATP in the polymerization mixture allows measurement of the net sum of DNA polymerization and ATP-based excision activities. (B) Time dependence of ATP-based rescue of TFV-terminated primers. (C) ATP-based rescue was dependent on concentration of ATP.
(PPTX)

Figure S3 Pre-steady state incorporation of dATP or TFV-DP by K70Q and Q151Mc HIV-1 RTs. Single-nucleotide incorporation of dATP (panels A, B, and E) or TFV-DP (panels C, D, and F) by K70Q (panels A, C, E, and F) and Q151Mc (panels B, D, E, and F). Formation of extended primer products in the reactions with K70Q RT and Q151Mc RT were measured at 5 ms to 5 s time points, using the following dATP concentrations: 0.5 (▪), 1 (□), 2.5 (▴), 5 (*), 10 (♦), 20 (◊), 50 (▾) and 75 µM (+). Incorporation of TFV was measured at 0.1–10 s reactions and at the following TFV-DP concentrations: 0.75 (▪), 1.5 (□), 3.75 (▴), 7.5 (*), 15 (♦), 37.5 (◊) and 75 µM (▾) for reactions with K70Q RT (panel C), and 3.75 (▴), 7.5 (*), 37.5 (◊), 55 (▾), 75 (+) and 112.5 (•) for reactions with Q151Mc RT (panel D). (E) The amplitudes of the burst phases from the dATP reactions shown in panels A (K70Q, [▴]) and B (Q151Mc, [▾]) were plotted as a function of dATP concentrations. (F) The amplitudes of the burst phases from the TFV-DP reactions shown in panels C (K70Q, [▴]) and D (Q151Mc, [▾]) were plotted as a function of TFV-DP concentrations. The solid lines in panels A, B, C, and D represent the best fit of data to a burst equation. Each point represents average values of three experiments.
(PPTX)

Table S1 Drug susceptibility of clinical isolates.
(DOC)

Table S2 Drug susceptibility of HIV-1 variants carrying mutation at residue 70.
(DOC)

Table S3 Drug susceptibility of HIV-1 variants carrying mutation at residue 70 in the background of Q151M complex.
(DOC)
