# Supplementary material for: K70Q Adds High-Level Tenofovir Resistance to “Q151M Complex” HIV Reverse Transcriptase through the Enhanced Discrimination Mechanism
Source: PLoS One. 2011 Jan 13;6(1):e16242. doi: 10.1371/journal.pone.0016242 (PMC3020970; doi:10.1371/journal.pone.0016242)
Supplement: Figure S1 — Amino acid sequence alignment of the RT regions (amino acid 32 to 560) of the clinical isolates at time points 1 to 2 (see Figure 1A). (DOC) [file pone.0016242.s001.doc]

GenBank accession number32 62 70 75 102 106 108 111

NL4-3 KALVEICTEM EKEGKISKIG PENPYNTPVF AIKKKDSTKW RKLVDFRELN KRTQDFWEVQ LGIPHPAGLK QKKSVTVLDV

1 AB506802 ---------- ---------- ---------- V-----G--- ---I------ --------I- ---------Q K---M-----

2 AB506803 ---------- ---------- ---------- V-----G-Q- ---I------ --------I- ---------Q R---M-I---

116 151 201

NL4-3 GDAYFSVPLD KDFRKYTAFT IPSINNETPG IRYQYNVLPQ GWKGSPAIFQ CSMTKILEPF RKQNPDIVIY QYMDDLYVGS DLEIGQHRTK

1 ----Y----- ES-------- ---T------ ---------M ---------- S--------- KSK----D-- ---------- --------K-

2 ----Y----- ES-------- ---T------ ---------M ---------- S--------- KSK----D-- ---------- --------K-

　 　 　　 291

NL4-3 IEELRQHLLR WGFTTPDKKH QKEPPFLWMG YELHPDKWTV QPIVLPEKDS WTVNDIQKLV GKLNWASQIY AGIKVRQLCK LLRGTKALTE

1 V--------K --L------- ---------- ---------- ---------- ---------- ---------- P--------- ----------

2 V--------K --L------- ---------- ---------- ---------- ---------- ---------- P--------- ----------

381

NL4-3 VVPLTEEAEL ELAENREILK EPVHGVYYDP SKDLIAEIQK QGQGQWTYQI YQEPFKNLKT GKYARMKGAH TNDVKQLTEA VQKIATESIV

1 ---------- ---------- ---------- ---------- ---------- ---------- -------R-- ---------- ----T-----

2 ---------- ---------- ---------- ---------- ---------- ---------- -------R-- ---------- ----T-----

471

NL4-3 IWGKTPKFKL PIQKETWEAW WTEYWQATWI PEWEFVNTPP LVKLWYQLEK EPIIGAETFY VDGAANRETK LGKAGYVTDR GRQKVVPLTD

1 ----I---R- --------T- -I-------- ---------- ---------- -------R-- --------I- ---------- ----------

2 ----I---R- --------T- -I-------- ---------- ---------- -------R-- --------I- ---------- ----------

560

NL4-3 TTNQKTELQA IHLALQDSGL EVNIVTDSQY ALGIIQAQPD KSESELVSQI IEQLIKKEKV YLAWVPAHKG IGGNEQVDGL VSAGIRKVL

1 ---------- ---------- ---------- ---------- ---------- -----N---- ---------- --------K- --N---R--

2 ---------- ---------- ---------- ---------- ---------- -----N---- ---------- --------K- --N---R--
