# Supplementary material for: K70Q Adds High-Level Tenofovir Resistance to “Q151M Complex” HIV Reverse Transcriptase through the Enhanced Discrimination Mechanism
Source: PLoS One. 2011 Jan 13;6(1):e16242. doi: 10.1371/journal.pone.0016242 (PMC3020970; doi:10.1371/journal.pone.0016242)
Supplement: Figure S2 — Effects of RT mutations K70Q, Q151Mc, or K70Q/Q151Mc on DNA primer extension activity and on ATP-based excision activities. (A) Effect of varying concentrations of TFV-DP on the primer extension activities of HIV-1 WT and mutant RTs. The experiments were carried out in the presence and absence of 3.5 mM ATP (marked as ATP (+) and ATP (−), respectively). Addition of ATP in the polymerization mixture allows measurement of the net sum of DNA polymerization and ATP-based excision activities. (B) Time dependence of ATP-based rescue of TFV-terminated primers. (C) ATP-based rescue was dependent on concentration of ATP. (PPTX) [file pone.0016242.s002.pptx]

## Slide 1
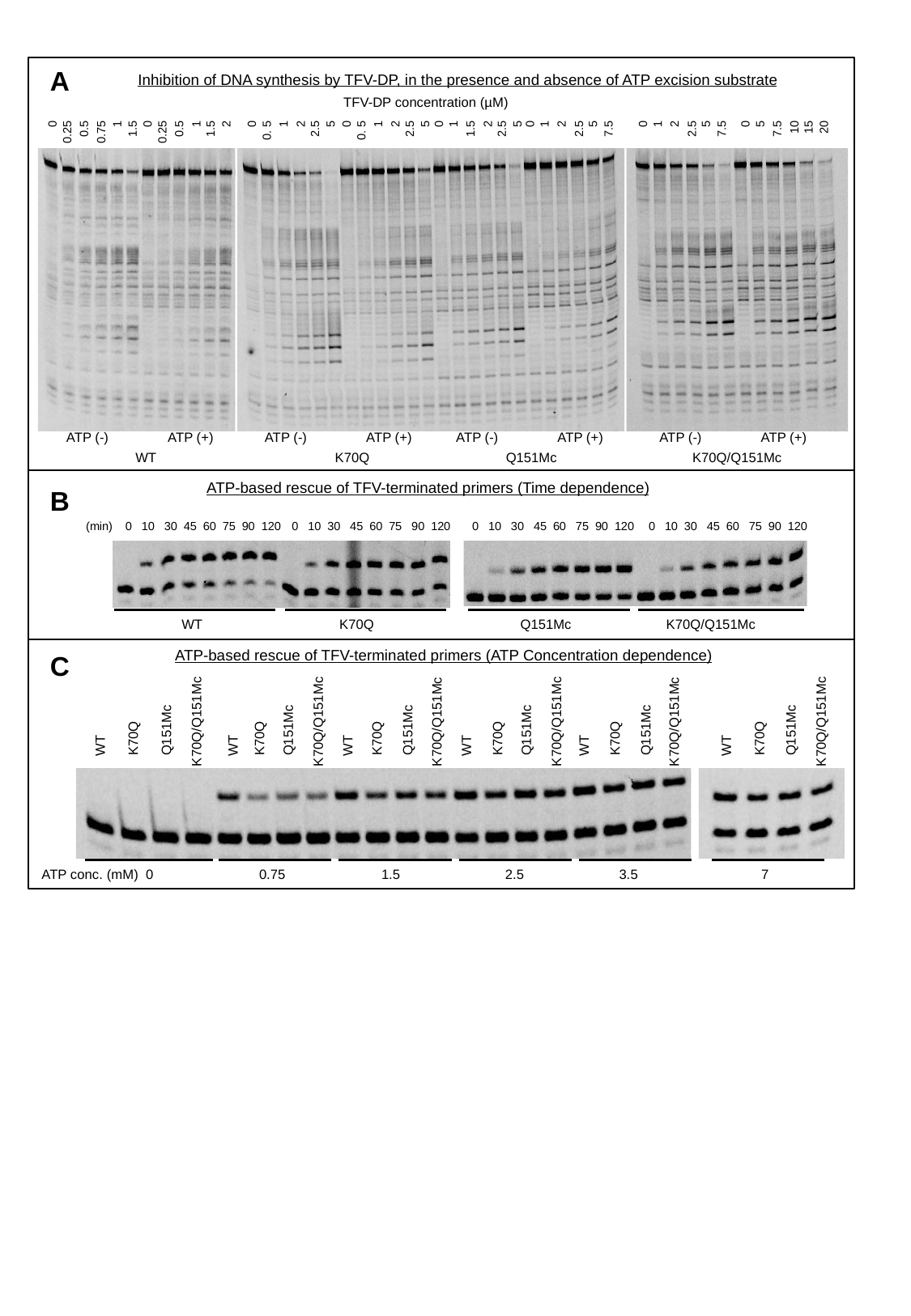

A
Inhibition of DNA synthesis by TFV-DP, in the presence and absence of ATP excision substrate
TFV-DP concentration (µM)
0
1
0
1
2
0.5
1.5
0.5
1.5
0.25
0.75
0.25
1
2
5
0
2.5
0. 5
0
1
2
5
2.5
0. 5
0
1
2
5
1.5
2.5
0
1
2
5
2.5
7.5
0
1
2
5
2.5
7.5
0
5
10
15
20
7.5
ATP (-)
ATP (+)
ATP (-)
ATP (+)
ATP (-)
ATP (+)
ATP (-)
ATP (+)
WT
K70Q
Q151Mc
K70Q/Q151Mc
ATP-based rescue of TFV-terminated primers (Time dependence)
B
(min) 0 10 30 45 60 75 90 120
 0 10 30 45 60 75 90 120
 0 10 30 45 60 75 90 120
 0 10 30 45 60 75 90 120
WT
K70Q
Q151Mc
K70Q/Q151Mc
ATP-based rescue of TFV-terminated primers (ATP Concentration dependence)
C
K70Q/Q151Mc
K70Q/Q151Mc
K70Q/Q151Mc
K70Q/Q151Mc
K70Q/Q151Mc
K70Q/Q151Mc
Q151Mc
Q151Mc
Q151Mc
Q151Mc
Q151Mc
Q151Mc
K70Q
K70Q
K70Q
K70Q
K70Q
K70Q
WT
WT
WT
WT
WT
WT
ATP conc. (mM) 0
0.75
1.5
2.5
3.5
7
