# Supplementary figures and images for: K70Q Adds High-Level Tenofovir Resistance to “Q151M Complex” HIV Reverse Transcriptase through the Enhanced Discrimination Mechanism
Source: PLoS One. 2011 Jan 13;6(1):e16242. doi: 10.1371/journal.pone.0016242 (PMC3020970; doi:10.1371/journal.pone.0016242)

## Slide 1
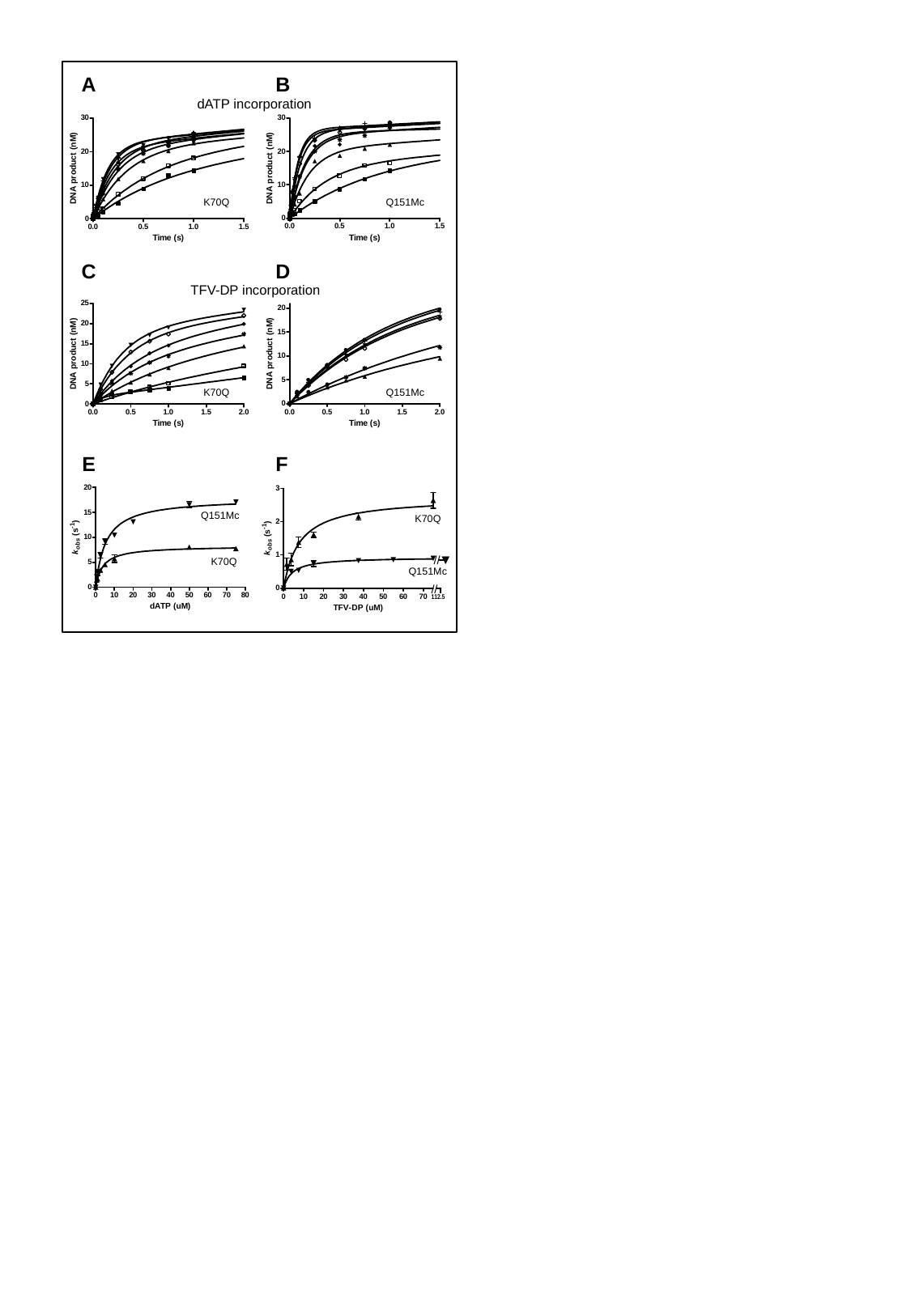

A
B
dATP incorporation
K70Q
Q151Mc
C
D
TFV-DP incorporation
K70Q
Q151Mc
E
F
K70Q
▼
//
Q151Mc
//
112.5
Q151Mc
K70Q

Supplement: Figure S3 — Pre-steady state incorporation of dATP or TFV-DP by K70Q and Q151Mc HIV-1 RTs. Single-nucleotide incorporation of dATP (panels A, B, and E) or TFV-DP (panels C, D, and F) by K70Q (panels A, C, E, and F) and Q151Mc (panels B, D, E, and F). Formation of extended primer products in the reactions with K70Q RT and Q151Mc RT were measured at 5 ms to 5 s time points, using the following dATP concentrations: 0.5 (▪), 1 (□), 2.5 (▴), 5 (*), 10 (♦), 20 (◊), 50 (▾) and 75 µM (+). Incorporation of TFV was measured at 0.1–10 s reactions and at the following TFV-DP concentrations: 0.75 (▪), 1.5 (□), 3.75 (▴), 7.5 (*), 15 (♦), 37.5 (◊) and 75 µM (▾) for reactions with K70Q RT (panel C), and 3.75 (▴), 7.5 (*), 37.5 (◊), 55 (▾), 75 (+) and 112.5 (•) for reactions with Q151Mc RT (panel D). (E) The amplitudes of the burst phases from the dATP reactions shown in panels A (K70Q, [▴]) and B (Q151Mc, [▾]) were plotted as a function of dATP concentrations. (F) The amplitudes of the burst phases from the TFV-DP reactions shown in panels C (K70Q, [▴]) and D (Q151Mc, [▾]) were plotted as a function of TFV-DP concentrations. The solid lines in panels A, B, C, and D represent the best fit of data to a burst equation. Each point represents average values of three experiments. (PPTX) [file pone.0016242.s003.pptx]
