# Supplementary material for: K70Q Adds High-Level Tenofovir Resistance to “Q151M Complex” HIV Reverse Transcriptase through the Enhanced Discrimination Mechanism
Source: PLoS One. 2011 Jan 13;6(1):e16242. doi: 10.1371/journal.pone.0016242 (PMC3020970; doi:10.1371/journal.pone.0016242)
Supplement: Table S1 — Drug susceptibility of clinical isolates. (DOC) [file pone.0016242.s004.doc]

| **Isolate^a^** | **EC_50_, (µM)** | | | | | | | | |
| --- | --- | --- | --- | --- | --- | --- | --- | --- | --- |
|  | **AZT** | **ddI** | **d4T** | **3TC** | **ABC** | **TFV-DF** | **FTC** | **NVP** | **EFV** |
| WT | 0.023 ± 0.002^b^ | 2.5 ± 0.35 | 2.0 ± 0.55 | 1.4 ± 0.15 | 1.6 ± 0.46 | 0.02 ± 0.003 | 0.4 ± 0.03 | 0.05 ± 0.004 | 0.002 ± 0.0001 |
|  |  |  |  |  |  |  |  |  |  |
| 1 | 1.4 ± 0.2 | 29.3 ± 3.06 | 18.3 ± 1.53 | 18.7 ± 2.08 | 16.3 ± 2.52 | 0.04 ± 0.01 | 3.5 ± 0.12 | >10 | >1 |
|  | **(61)^c^** | **(12)** | **(9.2)** | **(13)** | **(10)** | (2) | **(9)** | **(>200)** | **(>500)** |
| 2 | 1.3 ± 0.1 | >100 ± 0 | >100 ± 0 | 43 ± 1.73 | 24.7 ± 2.89 | 0.12 ± 0.02 | 2.1 ± 0.17 | >10 | >1 |
|  | **(57)** | **(>40)** | **(>50)** | **(31)** | **(15)** | **(6)** | **(5)** | **(>200)** | **(>500)** |

^a^. Isolates 1 and 2 refer to the time points indicated in Figure 1A, and whose phenotypic and genotypic profiles are shown in Figures 1B and 1C.

^b^. Data are means ± standard deviations from at least three independent experiments.

^c^. Fold increase compared to HIV-1_WT_ is shown in parentheses. Bold indicates an increase in fold increase value greater than 3-fold.
