# Supplementary material for: K70Q Adds High-Level Tenofovir Resistance to “Q151M Complex” HIV Reverse Transcriptase through the Enhanced Discrimination Mechanism
Source: PLoS One. 2011 Jan 13;6(1):e16242. doi: 10.1371/journal.pone.0016242 (PMC3020970; doi:10.1371/journal.pone.0016242)
Supplement: Table S2 — Drug susceptibility of HIV-1 variants carrying mutation at residue 70. (DOC) [file pone.0016242.s005.doc]

| **Mutation** | **EC_50_, (µM)** | | | | | |
| --- | --- | --- | --- | --- | --- | --- |
|  | **AZT** | **ddI** | **d4T** | **3TC** | **ABC** | **TFV-DF** |
| WT | 0.034 ± 0.004^a^ | 2.4 ± 0.26 | 2.6 ± 0.23 | 1.4 ± 0.16 | 1.8 ± 0.22 | 0.02 ± 0.004 |
|  |  |  |  |  |  |  |
| K70R | 0.11 ± 0.01 | 3.3 ± 0.29 | 4.3 ± 0.57 | 2.5 ± 0.46 | 2.8 ± 0.31 | 0.02 ± 0.002 |
|  | **(3.2)^b^** | (1.4) | (1.7) | (1.8) | (1.6) | (1) |
| K70G | 0.03 ± 0.003 | 16 ± 1.5 | 4.3 ± 0.26 | 26 ± 0.58 | 6.6 ± 0.29 | 0.02 ± 0.003 |
|  | (0.9) | **(6.7)** | (1.7) | **(19)** | **(3.7)** | (1) |
| K70E | 0.05 ± 0.003 | 13 ± 3.5 | 16 ± 2.7 | 16 ± 2.1 | 7.3 ± 0.12 | 0.02 ± 0.003 |
|  | (1.5) | **(5.4)** | **(6.2)** | **(11)** | **(4.1)** | (1) |
| K70T | 0.03 ± 0.001 | 8.9 ± 0.31 | 2.2 ± 0.17 | 4.3 ± 0.29 | 2.6 ± 0.21 | 0.02 ± 0.003 |
|  | (0.9) | **(3.7)** | (0.8) | **(3.1)** | (1.4) | (1) |
| K70N | 0.04 ± 0.001 | 3.7 ± 0.32 | 3.4 ± 0.25 | 2.2 ± 0.25 | 3.2 ± 0.3 | 0.02 ± 0.003 |
|  | (1.2) | (1.5) | (1.3) | (1.6) | (1.8) | (1) |
| K70Q | 0.05 ± 0.002 | 12 ± 1.7 | 3 ± 0.21 | 4.6 ± 0.53 | 3.5 ± 0.6 | 0.03± 0.003 |
|  | (1.5) | **(5)** | (1.2) | **(3.3)** | (1.9) | (1.5) |

^a^. Data are means ± standard deviations from at least three independent experiments.

^b^. Fold increase compared to HIV-1_WT_ is shown in parentheses. Bold indicates a greater than 3-fold increase.
