# Supplementary material for: K70Q Adds High-Level Tenofovir Resistance to “Q151M Complex” HIV Reverse Transcriptase through the Enhanced Discrimination Mechanism
Source: PLoS One. 2011 Jan 13;6(1):e16242. doi: 10.1371/journal.pone.0016242 (PMC3020970; doi:10.1371/journal.pone.0016242)
Supplement: Table S3 — Drug susceptibility of HIV-1 variants carrying mutation at residue 70 in the background of Q151M complex. (DOC) [file pone.0016242.s006.doc]

| **Mutation** | **EC_50_, (µM)** | | | | | |
| --- | --- | --- | --- | --- | --- | --- |
|  | **AZT** | **ddI** | **d4T** | **3TC** | **ABC** | **TFV-DF** |
| Q151Mc | 3.3 ± 0.4^a^ | 45 ± 1 | 25 ± 2.3 | 29 ± 3.2 | 15 ± 2.1 | 0.04 ± 0.003 |
|  |  |  |  |  |  |  |
| Q151Mc/K70R | 2.9 ± 0.21 | 53 ± 3.1 | 44 ± 4.5 | 16 ± 1.5 | 14 ± 1 | 0.03 ± 0.002 |
|  | (0.9)^b^ | (1.2) | (1.8) | (0.6) | (0.9) | (0.8) |
| Q151Mc/K70G | 3.2 ± 0.23 | 59 ± 5 | 115 ± 5.1^c^ | 46 ± 2.5 | 35 ± 2.7 | 0.04 ± 0.002 |
|  | (1) | (1.3) | **(4.6)** | (1.6) | (2.5) | (1) |
| Q151Mc/K70E | 6.2 ± 0.2 | 30 ± 1.7 | 32 ± 3.6 | 11 ± 1.4 | 34 ± 2.1 | 0.03 ± 0.001 |
|  | (1.9) | (0.7) | (1.3) | (0.4) | (2.3) | (0.8) |
| Q151Mc/K70T | 2.4 ± 0.15 | 13 ± 1.5 | 16 ± 2.7 | 12 ± 2.5 | 25 ± 2.3 | 0.05 ± 0.001 |
|  | (0.7) | (0.3) | (0.6) | (0.4) | (1.7) | (1.3) |
| Q151Mc/K70N | 1.3 ± 0.1 | 109 ± 4.4^c^ | 25 ± 4 | 15 ± 1.7 | 16 ± 2.1 | 0.06 ± 0.004 |
|  | (0.4) | (2.4) | (1) | (0.5) | (1.1) | (1.5) |
| Q151Mc/K70Q | 7 ± 0.76 | 108 ± 2.5^c^ | 110 ± 6.6^c^ | 81 ± 0.08 | 24 ± 2.9 | 0.2 ± 0.01^c^ |
|  | (2.1) | (2.4) | **(4.4)** | (2.8) | (1.6) | **(5)** |

^a^. Data are means ± standard deviations from at least three independent experiments.

^b^. Fold increase compared to HIV-1_Q151Mc_ is shown in parentheses. Bold indicates a greater than 3-fold increase.

^c^. These EC_50_ values have statistically significant differences (*P*<0.0001 by t-test) with the values for HIV-1_Q151Mc_.
